# Extent of Neck Dissection and Cervical Lymph Node Involvement in Oral Squamous Cell Carcinoma

**DOI:** 10.3389/fonc.2022.812864

**Published:** 2022-05-24

**Authors:** Philipp Thoenissen, Anja Heselich, Stefanie Deeg, Sarah Al-Maawi, Anna Tanneberger, Robert Sader, Shahram Ghanaati

**Affiliations:** ^1^ Clinic for Maxillofacial and Plastic Surgery, University Hospital Frankfurt, Goethe University, Frankfurt Am Main, Germany; ^2^ Frankfurt Oral Regenerative Medicine (FORM), Clinic for Maxillofacial and Plastic Surgery, University Hospital Frankfurt, Goethe University, Frankfurt Am Main, Germany

**Keywords:** neck dissection (ND), OSCC (oral squamous cell carcinoma), cervical metastasis, follow up, MRND (modified radical neck dissection), SND (selective neck dissection)

## Abstract

**Introduction:**

Tumor resection combined with neck dissection (ND) or radiotherapy are established methods for the treatment of patients with oral squamous cell carcinoma (OSCC). However, the extent of ND can lead to postoperative complications. Therefore, for the first time, this study aims to identify lymph node involvement in OSCC performed in a bilateral systematic approach based on oncologic board meetings relying on presurgical magnetic resonance imaging (MRI) and computed tomography (CT).

**Materials and Methods:**

In a retrospective single-center study, patients with primary OSCC resection and systematic ND performed in 4 different manners (MRND III bilateral, MRND III left and SND right, MRND III right, SND left, and SND bilateral) were examined. Lymph node involvement allocated to levels was evaluated depending on primary localization and T-stage.

**Results:**

A total of 177 consecutive patients (mean age 63.64; 92 female, male 85) were enrolled in this study. A total of 38.98% showed cervical lymph node involvement, and metastases were found in levels 1–4. The distribution of positive lymph node metastases (n=190 LNs) was 39.47% in level 1, 38.95% in level 2, 10.53% in level 3, and 11.05% in level 4.

**Discussion:**

In a cohort of OSCC patients with systematic bilateral ND, levels 1 and 2 had positive lymph node involvement, and no lymph node involvement was seen at level 5. Without any clinical or imaging suspicion, ND expanding 5-level MRND should be avoided regardless of the primary tumor localization, T-stage and intraoperative proof of cervical metastases.

## Introduction

According to the current guidelines, primary tumor resection and neck dissection (ND) are established methods for healing and prolonging the life of patients with oral squamous cell carcinoma (OSCC). A therapy recommendation is made after discussing the patient case in an interdisciplinary oncologic board meeting. The verdict is based on a histological examination of the malignant tumor as well as numerous staging examinations leading to a preoperative classification of TNM and UICC classification. These include clinical examination and imaging procedures such as orthopantomography/panorex (OPG), ultrasound, computed tomography (CT) and magnetic resonance imaging (MRI). Ultrasound, CT and MRI are important for diagnostics in cervical lymph node involvement of oral squamous cell carcinoma (1). Especially with MRI, a high specificity for the assessment of cervical lymph node metastases is possible (2, 3). In addition to tumor resection, surgical therapy comprises ND, which is possible in many variants. Additionally, radiotherapy is often conducted as an adjuvant therapy option. The indication for adjuvant radiation therapy depends on several factors, i.e., status of resection or particular nodal status (4, 5). Consequently, the findings after lymph node removal are of eminent importance for the proposal of any radiation therapy.

The extent of cervical lymph node removal is dependent on the clinical and imaging classification of cervical metastasis, the “T” stage, and the localization of the primary tumor. In Germany, the recommendation is based on the national guidelines for tumors of the oral cavity (1). Lymph node metastases are seen as the most important prognostic factor (6).

ND is differentiated into elective and therapeutic. Elective ND is performed in case there is no clinical hint for lymph node involvement. Therapeutic ND is conducted in cases of clinical evidence for lymph node involvement at first diagnosis or in cases of nodal relapse. The latter is also called salvage ND (7). This classification is independent of the extent of ND. The extent of ND is determined by anatomical regions introduced by Robbins as a general classification on the topography of 6 levels of cervical lymph nodes. The levels are defined within anatomical structures of the head and neck area (see **Table 1**). According to this classification, 4 types of ND have been described: radical neck dissection (RND), modified radical neck dissection (MRND), selective neck dissection (SND), and extended neck dissection (END) (8, 9) (**Table 2**).

**Table 1:** Levels and their boundaries according to Robbins classification (8).

Level	Name and boundaries
Level IA	Submental (anterior: symphysis, inferior: hyoid, medial: anterior belly of contralateral digastric muscle, lateral: anterior belly of ipsilateral digastric muscle)
Level IB	Submandibular (mandible; posterior belly of muscle; anterior belly of digastric muscle; stylohyoid muscle)
Level II A and B	Upper jugular nodes (skull base; inferior body of the hyoid bone; stylohyoid muscle/vertical plane of the spinal accesory nerve; vertical plane of the spinal accesory nerve/lateral border of the sternocleidoid muscle)
Level III	Middle jugular group (inferior body of hyoid; inferior border of cricoid cartilage; lateral border of sternohyoid muscle; lateral border of sternocleidoid muscle or sensory branches of cervical plexus)
Level IV	Lower jugular group (inferior border of the cricoid cartilage; clavicle; lateral border of sternohyoid muscle; lateral border of sternocleidoid muscle or sensory branches of cervical plexus)
Level VA and B	Posterior triangle group (apex of convergence of sternocleidoid and trapezius muscle/lower border of cricoid cartilage; lower border of the cricoid cartilage/clavicle; posterior border of sternocleidoid muscle or sensory branches of cervical plexus; anterior border of trapezius muscle (7)
Level VI	Anterior compartment group (hyoid bone; suprasternal; common carotid artery; common carotid artery)

**Table 2:** Types of ND according to Robbins and Medina.

Types of neck dissection (ND)	Extent
Radical neck dissection (RND)	lymphatic structures and 1 or more of SAN, IJV, SCM
Selective neck dissection (SND)	defined levels (I to III, I to IV) of lymphatic structures without other structures
*Lateral/anterolateral/posterolateral*	
Modified radical neck dissection (MRND)	
*MRND I*	RND without SAN
*MRND II*	without SAN, IJV
*MRND III ("functional ND")*	without SAN, IJV, SCM
Extended neck dissection	RND with one or more lymphatic or other anatomical structures

SAN, Spinal accessory nerve; IJF, internal jugular vein; SCM, sternocleido muscle; MRND, modified radical neck dissection; SND, selective neck dissection.

For OSCC, elective ND is recommended due to occult metastases that are present in approximately 20% of patients without clinical and radiological suspicion of cervical lymph node involvement (10). Conversely, it is known that the extent and radicality of ND increases postoperative complications (11, 12). These include restricted movement of the neck and shoulders, nerve damage, and bleeding complications. To reduce complications, the radical has now been abandoned in favor of modified ND.

Some data exist about the rate and extent of lymph node metastases in OSCC (13–16). These data, however, include OSCC not solely of the oral cavity and show either no or a limited collection of primary localization. Others do not give any exact information about the types of ND performed ipsi- and bilaterally.

Accordingly, for the first time, the present study intends to depict the occurrence of lymph node involvement of a single center observation on a specific systematic and reproducible bilateral approach based on an interdisciplinary oncologic board meeting relying on magnetic resonance imaging (MRI) and computed tomography (CT).

## Materials and Methods

A review of all patients with histopathological proof of OSCC and primary surgery in the Department of Craniomaxillofacial and Plastic Surgery, University Medical Center Frankfurt, Germany, from January 1, 2014 until December 31, 2020 was conducted. Patients were identified by analyzing internal databases and confirmed manually. Ethics approval was given (03/2013; 40/18; 2021–76). The study has been registered in the German Clinical Trials Register (DRKS) Number 00016654.

The inclusion criteria were first diagnosis histopathological proof of OSCC and operable primary manifestation in treatment naïve patients, bilateral SND (level I to IV), MRND III (level I to V) or a combination of those. The following parameters were recorded and evaluated: age, sex, anatomical localization of the tumor, TNM classification as T-stage, grading, lymph node involvement, and lymph node involvement according to levels of Robbins classification.

Patients were excluded if they had other malignancies, extraoral localization, primary nonsurgical intervention such as radiotherapy or a combination of radio and oncologic treatment.

All patients received CT or MRI of the head and neck for staging in addition to clinical examinations. Surgery was comprised of primary resection of the tumor and ND. A decision was made based on the preoperative findings, a demonstration of radiological imaging, and an oncologic board recommendation comprised of a craniomaxillofacial surgeon, radiologist, otolaryngologist, radiotherapist, oncologist, and pathologist. ND was performed as anterolateral SND including level I to IV or MRND III including levels I to V without resection of sternocleidomastoid muscle, the internal jugular vein, and/or the XI nerve according to Medina. The following approach was chosen in cases with a primary decision for a ND of both sides, which was indicated when midline involvement of the primary tumor or bilateral lymph node involvement was detected; initially anterolateral SND was performed bilaterally. In cases of clinically and/or histopathologically lymph node involvement proven by frozen sections during surgery, anterolateral SND of the affected side was expanded to ipsilateral MRND III. Clinical hints for involved lymph nodes were set to a 1.5 cm diameter or degradation of the lymph nodes capsula.

The following procedure was performed in cases with a decision for an ND of one side: initially, anterolateral SND was performed unilaterally. In cases of histopathologically proven lymph node involvement during surgery, the anterolateral SND of the affected side was expanded to ipsilateral MRND III, while the contralateral side underwent an anterolateral SND. Additionally, here, an expansion to an MRND III was performed in case of proven lymph node involvement.

Therefore, 4 groups of ND types and combinations were defined: MRND III bilateral (level I to V); MRND left (level I to V), SND right (level I to IV); MRND III right (level I to V), SND left (level I to IV); and SND bilateral (level I to IV).

Resected tissue was sent for histopathological examination, which was then performed in a routine manner.

Nine different localizations of the primary tumor were defined: lower lip, floor of mouth (FOM), alveolar process of upper jaw, alveolar process of lower jaw, tongue, buccal, soft palate, hard palate, and retromolar region.

Follow-up period has a range from 3 to 63 months.

All patients’ cases have been discussed after surgery with the definite histopathological results. Depending on these results, follow-up or adjuvant radio or radiochemotherapy, according to the current German guidelines have been administered (1).

**Figure 1:**
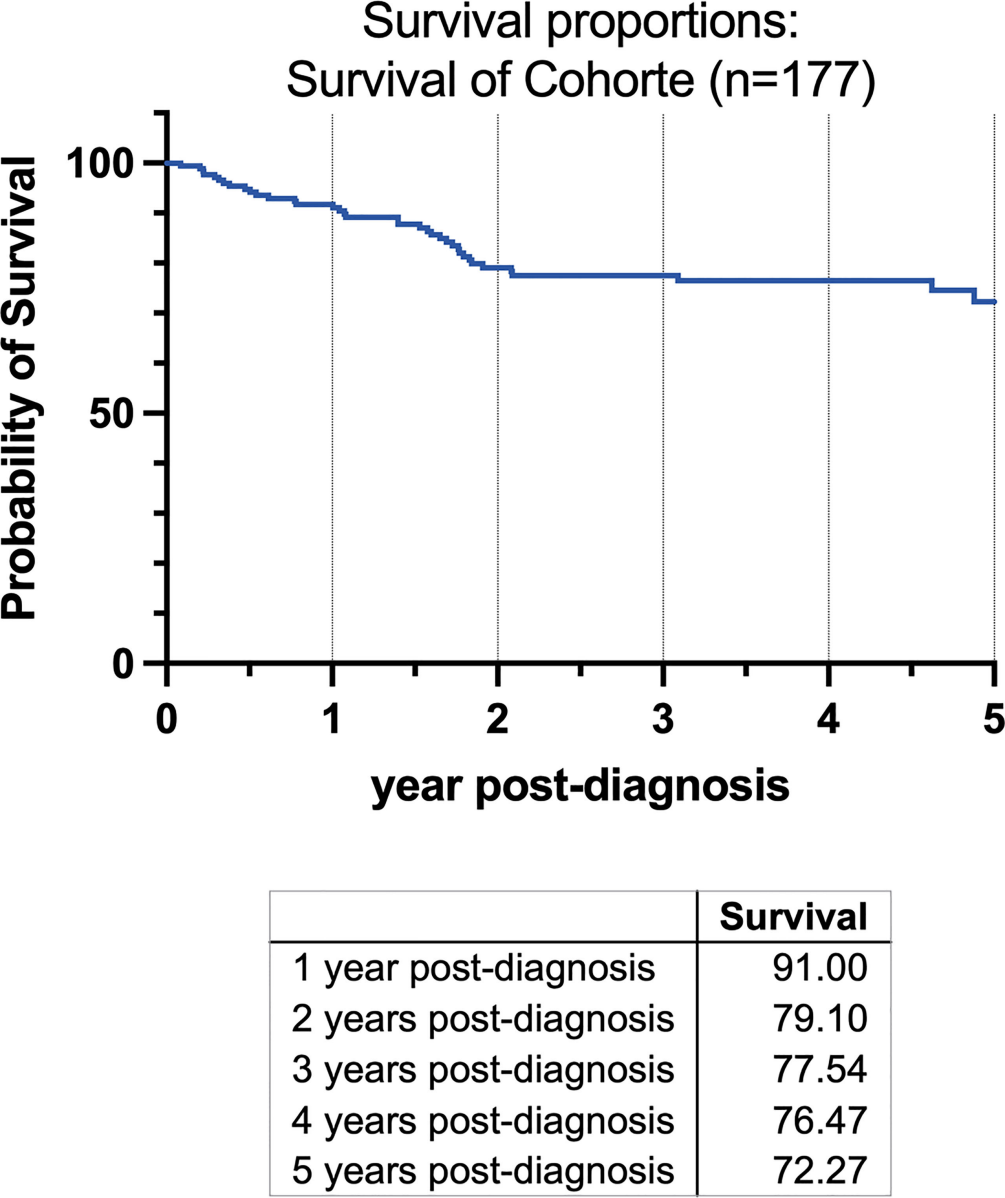
Kaplan-Meier-Graph of all patients for 5 years beginning at the time of the initial diagnosis. Survival in the first year was 91%, 79.1% in the second year after diagnosis, 77.54% in third, 76.47% in fourth and 72.72% after five years.

A survival analysis has been conducted and was depicted in a Kaplan-Meier-Graph (**Figure 1**). As no statistical relevant information could be gained by dividing the cohort into subgroups, a graph was plotted for the complete cohort of patients (n=177).

All statistical analyses were performed using Prism 8 (GraphPad, USA). Descriptive statistics for quantitative variables are given as the mean value.

## Results

The study reviewed a cohort of patients with primary tumor resection of oral squamous cell carcinoma (OSCC) with systematic bilateral neck dissection (ND).

### Patient Collective

A total of 177 consecutive patients (92 women, 85 men) who fit the inclusion criteria with a mean age of 63.64 years (SD 11.98, range 29-92 y), female 63.32 years (SD 11.98, 29–92 y), male 62.45 years (SD 10.47; 37–87 y) were included in this study (**Table 3**). A total of 49.63 (SD 22.79, min 9, max 142) lymph nodes were dissected on average per patient. Survival was 91% in the first year after diagnosis, 79.1% in the second year, 77.54% in third year, 76.47% in the fourth year, and 72.27% after the fifth year (**Figure 1**).

**Table 3:** Patient and age distribution of the patient cohort.

	Female	Male
Frequency	92	85
Mean Age	64.75	62.45
Minimum	29	37
Maximum	92	87
Std. Deviation	13.14	10.47

### Tumor Stage

All resected tumors were histopathologically analyzed and classified in all cases as oral squamous cell carcinoma (OSCC). Tumors were characterized according to T-size (**Table 4**), lymph node involvement (**Table 5**), and grading (1–3; data not shown). The T-size distribution of the primary tumors ranged from T1 to T4 (T1 = 54 (30.51%), T2 = 53 (29.94%), T3 = 37 (20.91%), T4 = 43 (24.29%)).

**Table 4:** pT-stage for primary diagnosis in the whole patient cohort.

	Frequency	Percent
T1	54	30.51
T2	53	29.94
T3	37	20.90
T4	33	18.64

Data are represented as total and relative values, based on n=177 patients.

**Table 5:** Number of cervical lymph node metastases distributed according to dissected levels.

	Frequency	Percent
Level 1	75	39.47
Level 2	74	38.95
Level 3	20	10.53
Level 4	21	11.05

The majority of lymph node metastases were found in Level 1 (39.47%) and Level 2 (38.95%). Only a total of 20 lymph node metastases were found in level 3 (10.53%) and 21 in level 4 (11.05%). No lymph node metastases were found at other dissected levels. Data are represented as total values based on n=190 LN metastases in n=177 patients.

Primary localization of the tumor was divided into nine groups, anatomically defined from front to retromolar region: lower lip, floor of mouth (FOM), alveolar process of upper jaw, alveolar process of lower jaw, tongue, buccal, soft palate, hard palate, and retromolar region.

**Figure 2:**
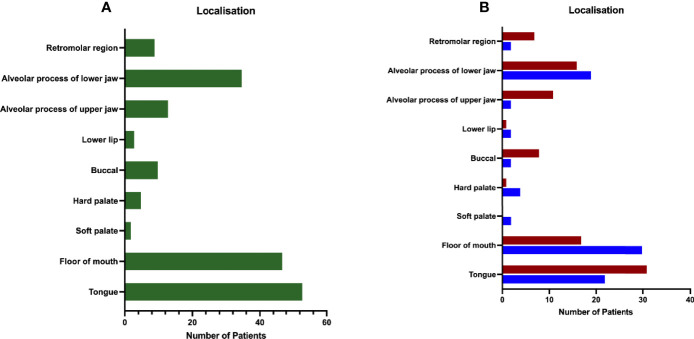
Distribution of patients by primary site. **(A)** The majority of the patient collective was diagnosed with primary tumor in tongue (33.70%), floor of mouth (FOM; 26.55%), and the alveolar process of the lower jaw (19.77%), followed by tumors located in the buccal (5.65%) and retromolar (5.08%) regions, hard palate (2.82%), alveolar process of the upper jaw (7.34%), the lower lip (1.69%), and the soft palate (1.13%). **(B)** Distribution of primary tumor site according to gender shows two times more tumors for FOM in male patients (m: 35.29%/f: 18.48%), whereas in female patients more were diagnosed with tumors located in the buccal (m: 2.35%/f: 8.70%) or retromolar region (m: 2.35%/f: 7.61%). Data are presented as total values, based on n=177 patients.

The distribution of primary tumor localization is shown in **Figure 2A** for all patients and in **Figure 2B** differentiated by sex. The highest occurrence was observed in the tongue (53 cases, 29.94%) and FOM (47 cases, 26.55%), followed by the alveolar process of the lower jaw (35 cases, 19.77%). Lower frequencies were found for other locations: alveolar process of upper jaw (13 cases, 7.34%), buccal (10 cases, 5.65%), retromolar region (9 cases, 5.08%), hard palate (5 cases, 2.82%), lower lip (3 cases, 1.69%), and soft palate (2 cases, 1.13%). **Figure 2B** shows high discrepancy between genders concerning localization of FOM, with the nearly double amount in men than in women.

In 54 of all cases (30.51%), the tumor exceeded the midline and was present on both sides.

### Types of ND

**Figure 3:**
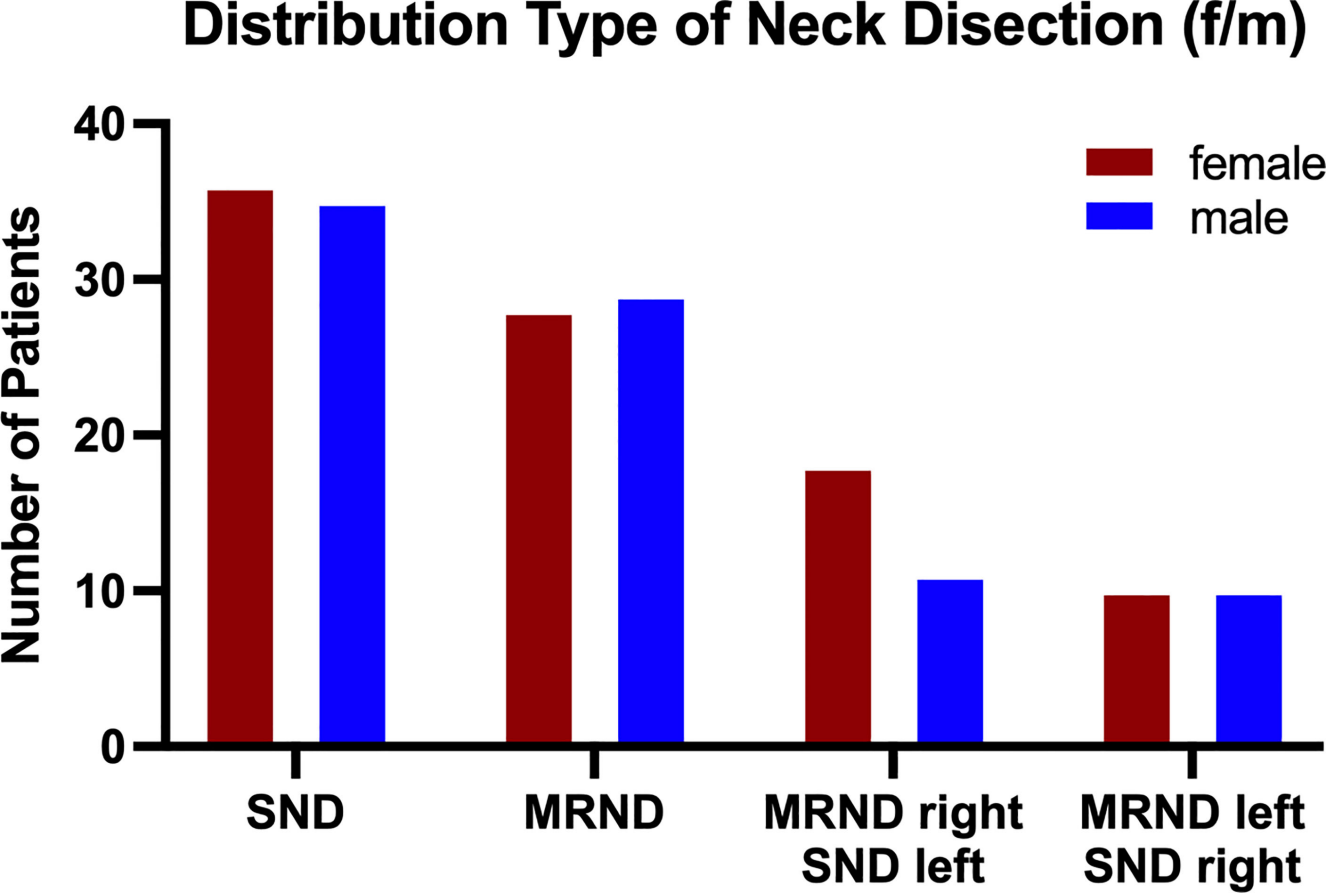
Population distribution by type of neck dissection. Neck dissection was performed *via* 4 different defined methods: (i) SND (I-IV) bilateral, (ii) MRND III (I-V) left/SND (I-IV) right, (iii) MRND III (I-V) right/SND (I-IV) left, and (iv) MRND III (I-V) bilateral. In 41.18% of all patients, SND bilateral was performed, followed by 34.12% dissected *via* MRND III, bilateral, 12.94% *via* MRND III, right/SND left, and 11.76% *via* MRND III, left/SND right. The gender distribution in all treatments was rather equal, except for MRND III, which was bilateral, with more male patients undergoing this treatment. Data are presented as total values, based on n=177 patients.

ND was performed in 4 different manners (as previously defined in “Materials and Methods”). The distribution of ND performed is shown in **Figure 3**, with a total distribution of SND bilateral=71 cases (40.11%), MRND III bilateral=57 cases (32.2%), MRND III right, SND left=29 cases (16.38%), MRND III left and SND right=20 cases (11.3%).

### Lymph Node Metastases

Most patients showed no lymph node involvement (108 cases, 61.02%). In the remaining patient cohort (69 cases, 38.98%), up to 11 lymph node metastases in one patient were found, with no significant differences between sexes. Most patients with lymph node metastasis showed 1–2 lymph nodes with metastasis. Fifteen (8.47%) patients had bilateral cervical metastases. Lymph node metastases were found in levels 1–4. The distribution of positive lymph nodes (total 190 LNs) showed 75 metastases at level 1 (39.47%), 74 at level 2 (38.95%), 20 at level 3 (10.53%), and 21 at level 4 (11.05%) (**Table 5**). In the subgroup of metastases in level 4, the tongue was the origin localization in 71%, followed by FOM with 19%, hard palate and alveolar process of the upper jaw with each 5%.

In 9% of all node positive patients, skip metastases were found in level 2, 3.38% had skip metastases in level 3 and 1.13% had skip metastases in level 4.

Hence, 78.48% of all positive lymph node metastases were found up to level 2. **Table 6** lists the exact pN-classification.

**Table 6:** pN-classification of all patients.

	Frequency	Percent
N0	108	61.01
N1	22	12.43
N2	1	0.6
N2b	22	12.43
**N2c**	12	6.78
**N3**	1	0.6
**N3b**	11	6.21

Data are represented as total and relative values, based on n=177 patients.

**Figure 4:**
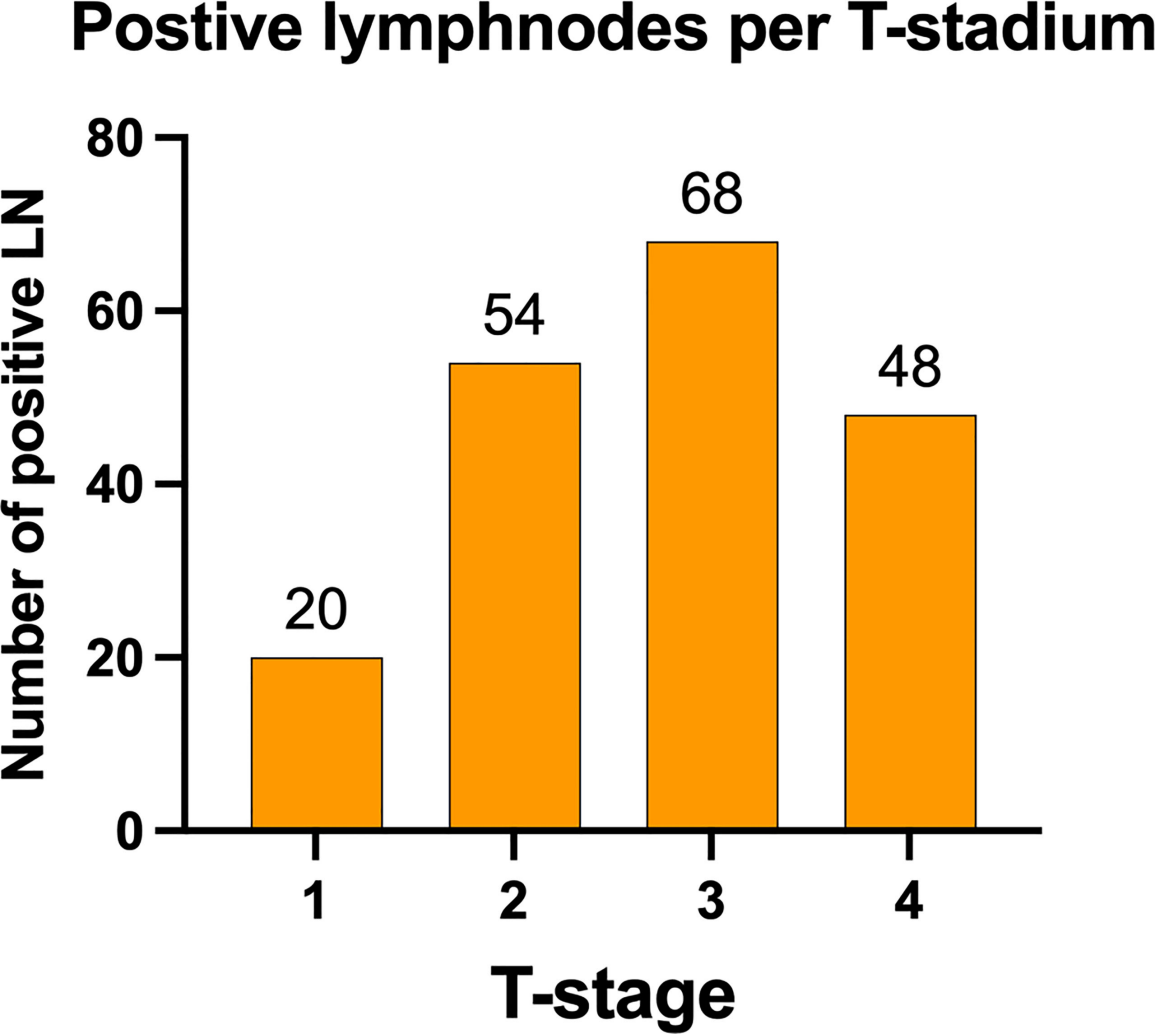
Distribution of positive lymph nodes according to tumor stage. Lymph node metastasis was predominately found in patients with primary tumors staged T2 and higher, with an almost equal distribution between T2 (28.42%), T3 (35.79%), and T4 (25.26%). Only 10.53% lymph node metastasis were found in T1 primary tumors. Data are presented as total values based on n=190 LN metastases in n=177 patients.

The distribution of lymph node metastases by tumor size showed mainly positive lymph nodes in higher T-stages T2-T4 with decreasing numbers with higher T-stages T2 = 54 (28.42%), T3 = 68 (35.79%), and T4 = 48 (25.26%). Only 20 lymph node metastases were found in T1 tumors (10.53%) (**Figure 4**). Per T1, 0.81% of all dissected nodes were positive; per T2, 2.19%; per T3, 3.20%; and for T4, 2.77%.

## Discussion

The present study was designed to retrospectively investigate the population of patients with oral squamous cell carcinoma (OSCC) of a German university hospital in the location of lymph node involvement after systematic bilateral neck dissection (ND). To the best of our knowledge, this investigation is the first based on a systematic bilateral approach based on the recommendation of an interdisciplinary oncologic board meeting relying on magnet resonance imaging (MRI) and computed tomography (CT). This is to provide an indication of the extent of ND of OSCC and possible adjuvant radiation therapy.

A total of 38.98% of the examined patients had histologically confirmed lymph node involvement. The study shows that cervical lymph node involvement in primarily surgically treated OSCC with concomitant ND is extended to levels 1 to 4. In total, 78.48% of tumor-positive lymph nodes were found in levels 1 and 2. Additionally, 11.05% of patients in level 4 showed lymph node involvement. No lymph node involvement was found in level 5.

Concerning the overall rate of cervical lymph node involvement, the results of the study are similar to those in the literature. Sagheb et al., retrospectively reviewed a German cohort of 204 patients with early stage T1 and T2 tumors of the tongue alone and found a rate of lymph node involvement of 23% (13). Moratin et al., found a rate of 42.6% for carcinoma in the mandible and 35.3% for the maxilla at initial diagnosis (17, 18).

Shah et al., examined a cohort of 501 patients with 516 cervical lymph node dissections in 1965 to 1986 with OSCC under radical ND. They found levels 1 to 3 to be major sites of lymphogenic metastasis (14). Kakei et al., reported positive lymph nodes in 100 patients with cN1 neck undergoing unilateral supraomohyoid ND regularly up to level 3, concerning tongue cancer also including level 4 (19). In particular, the results concerning the tongue as the primary localization of the tumor were similar to those of the present study.

Wharshawsky et al., 2019, postulated that for the clinical cN0 neck situation on the grounds of a meta-analysis, SND is adequate (20). Woolgar 1997, examined the localization of lymph node involvement depending on the localization of OSCC in 154 patients. Of these, 73 patients had a total of 347 tumor-positive lymph nodes; 20% of them were found in level 1, 47% in level 2, 16% in level 3, 13% in level 4, and 3% in level 5 (15). Although the extent of involvement in level 1 and level 2 is lower than that in the underlying study, the results also indicate that mainly the upper levels are affected by cervical metastases.

However, a general recommendation for only a 3-level ND based on these findings might be dangerous. De Zinis et al., found in a retrospective study of 89 otolaryngology department patients with bilateral complete ND occult or skip metastases in levels 3, 4 and 5 (21). In a large-scale study from 2015, D’Cruz highlighted the special importance of elective cervical lymph node dissection, which provides a survival advantage in T-stadiums 1 and 2 (7). However, this study relies only on an ultrasound scan of the neck after randomization and is thus inferior to general cervical objective and reproducible staging performed with CT or MRI.

Medina proposes to name any individual kind of ND by the parts additionally removed (22). For general comparability, it is important to define these subgroups. The patients included in the present study underwent only two different and defined forms of ND: SND and MRND. However, more subgroups make a statistical analysis difficult. Werner – as an otolaryngologist – points out that a comprehensive examination could only be carried out on the basis of uniform standards such as bilateral RND (23). Conversely, the case numbers for RND are too small to be inferred because radicality and patient impairment prohibit this form of ND today.

In addition, it can be assumed that imaging diagnostics as a therapy-critical precondition for surgical intervention ensures objective findings and high accuracy in the prediction of cervical lymph node involvement. The patients observed in the study invariably received local staging by MRI or CT examination. Ultrasound as an investigator-dependent method was not considered in the routine workflow of the clinic (24). In contrast to comparable studies, the surgery is also based on decisions of an interdisciplinary oncologic board meeting, with the result that precise therapy and extent of ND can be assumed preoperatively.

The aim of every therapeutic decision on the extent of ND should be, in addition to tumor-free survival, the least possible postoperative restrictions and complications. The results of the present study are, therefore, of particular interest as postoperative complications or limitations depend directly on the extent of ND (12). For example, the larger the anatomical area, the greater the risk of nerve injury and restriction of movement (25, 26). Recently, Ferreli et al., discovered that the amount of isolated lymph node metastases in level IIb is low. Accordingly, sparing level IIb is associated with a potentially lower risk of any impairment of the spinal accessory nerve (27) which is also involved in level V neck dissection. Thus, the extent to an ipsilateral MRND including level 5 without pathological evidence of metastasis should be questioned in order to improve postoperative quality of life.

The same holds true concerning the role of adjuvant radiation therapy; according to the high-risk factors such as positive margins and/or extracapsular nodal extension, adjuvant postoperative radiotherapy should be delivered. Lymphovascular invasion, perineural invasion, positive nodes, and Stadium T3 and 4 count for intermediate risk (5). Adjuvant radiotherapy should be applied in cases of positive margins, extranodal extension, T3 or T4, N2 and N3 and may be applied in cases of N1 without extranodal extension and adjusted to patients’ individual parameters and preferences (5). In the case of adjuvant radiotherapy, attention should be directed to the extent of the radiation field: a general recommendation for adjuvant radiation therapy with 54 Gy, an additional 12 Gy for special cases, is made for all draining lymph nodes at risk and dissected lymph node regions (4). However, there is still no sufficient and detailed definition for the included levels according to Robbins for these areas.

## Conclusion

In a cohort of 177 OSCC patients with systematic bilateral ND, levels 1 and 2 had positive lymph node involvement and no lymph node involvement was seen at level 5. Without any clinical or imaging suspicion, ND beyond level 4 and expanding to level 5 should be avoided regardless of the primary localization and T-stage of the tumor. Additionally, under the circumstances of finding positive lymph nodes intraoperatively, a standard approach for the extent to ipsilateral MRND should be prevented. Analogously, the extent of the radiation field in adjuvant radiation therapy needs to be questioned and limited when there is no sign of lymph node involvement in the primary staging.

## Data Availability Statement

The raw data supporting the conclusions of this article will be made available by the authors, without undue reservation.

## Ethics Statement

The studies involving human participants were reviewed and approved by Ethikkommission des Fachbereichs Medizin Universitätsklinikum der Goethe-Universität Theodor-Stern-Kai 7 Haus 1, 2. OG, Zimmer 207-211 60590 Frankfurt am Main. Written informed consent for participation was not required for this study in accordance with the national legislation and the institutional requirements.

## Author Contributions

PT and SG developed the idea for this project. PT and AH performed the statistical analysis. PT, AH, and SD performed the data collection. PT, AH, SA-M, AT, RS, and SG wrote and edited the manuscript. Each author contributed important content-related aspects. All authors contributed to the article and approved the submitted version.

## Conflict of Interest

The authors declare that the research was conducted in the absence of any commercial or financial relationships that could be construed as a potential conflict of interest.

## Publisher’s Note

All claims expressed in this article are solely those of the authors and do not necessarily represent those of their affiliated organizations, or those of the publisher, the editors and the reviewers. Any product that may be evaluated in this article, or claim that may be made by its manufacturer, is not guaranteed or endorsed by the publisher.
